# Study of the antitumor mechanisms of apiole derivatives (AP-02) from *Petroselinum crispum* through induction of G0/G1 phase cell cycle arrest in human COLO 205 cancer cells

**DOI:** 10.1186/s12906-019-2590-9

**Published:** 2019-07-27

**Authors:** Kuan-Hsun Wu, Wen-Jui Lee, Tzu-Chun Cheng, Hui-Wen Chang, Li-Ching Chen, Chia-Chang Chen, Hsiu-Man Lien, Teng-Nan Lin, Yuan-Soon Ho

**Affiliations:** 1 The Ph.D. Program for Translational Medicine, College of Medical Science and Technology, Taipei Medical University and Academia Sinica, Taipei, Taiwan; 2 Department of Pediatrics, School of Medicine, College of Medicine, Taipei Medical University, Taipei, Taiwan; 3 Department of Pediatrics, Wan Fang Hospital, Taipei Medical University, Taipei, Taiwan; 4 Ph.D. Program for Neural Regenerative Medicine, College of Medical Science and Technology, Taipei Medical University and National Health Research Institutes, Taipei, Taiwan; 5 School of Medical Laboratory Science and Biotechnology, College of Medical Science and Technology, Taipei Medical University, No. 250, Wu-Hsing Street, Taipei, 110 Taiwan, Republic of China; 6 Department of Medical Laboratory, and Cancer Research Center of Taipei Medical University Hospital, Taipei, Taiwan; 7 Division of Breast Surgery, Department of Surgery, Taipei Medical University Hospital, Taipei, Taiwan; 8 Taipei Cancer Center, Taipei Medical University, Taipei, Taiwan; 9 TMU Research Center of Cancer Translational Medicine, Taipei Medical University, Taipei, Taiwan; 10 School of Management, Feng Chia University, Taichung, Taiwan; 11 Research Institute of Biotechnology, Hungkuang University, No.1018, Sec. 6, Taiwan Blvd., Shalu Dist, Taichung City, 43302 Taiwan; 12 Institute of Biomedical Sciences, Academia Sinica, Taipei, Taiwan

**Keywords:** Apiole, COLO 205, Antitumor, *Petroselinum crispum*, G0/G1 arrest

## Abstract

**Background:**

Apiole was isolated from the leaves of various plants and vegetables and has been demonstrated to inhibit human colon cancer cell (COLO 205 cells) growth through induction of G0/G1 cell cycle arrest and apoptotic cell death. This study further explored the antitumor effects of apiole derivatives AP-02, 04, and 05 in COLO 205 cancer cells*.*

**Methods:**

Human breast (MDA-MB-231, ZR75), lung (A549, PE089), colon (COLO 205, HT 29), and hepatocellular (Hep G2, Hep 3B) cancer cells were treated with apiole and its derivatives in a dose-dependent manner. Flow cytometry analysis was subsequently performed to determine the mechanism of AP-02-induced G0/G1 cell cycle arrest. The in vivo antitumor effect of AP-02 (1 and 5 mg/kg, administered twice per week) was examined by treating athymic nude mice bearing COLO 205 tumor xenografts. The molecular mechanisms of AP-02-induced antitumor effects were determined using western blot analysis.

**Results:**

AP-02 was the most effective compound, especially for inhibition of COLO 205 colon cancer cell growth. The cytotoxicity of AP-02 in normal colon epithelial (FHC) cells was significantly lower than that in other normal cells derived from the breast, lung or liver. Flow cytometry analysis indicated that AP-02-induced G0/G1 cell cycle arrest in COLO 205 cells but not in HT 29 cells (< 5 μM for 24 h, **p < 0.01). Tumor growth volume was also significantly inhibited in AP-02 (> 1 mg/kg)-treated athymic nude mice bearing COLO 205 tumor xenografts compared to control mice (*p < 0.05). Furthermore, G0/G1 phase regulatory proteins (p53 and p21/Cip1) and an invasion suppressor protein (E-cadherin) were significantly upregulated, while cyclin D1 was significantly downregulated, in AP-02-treated tumor tissues compared to the control group (> 1 mg/kg, *p < 0.05).

**Conclusions:**

Our results provide in vitro and in vivo molecular evidence of AP-02-induced anti-proliferative effects on colon cancer, indicating that this compound might have potential clinical applications.

**Electronic supplementary material:**

The online version of this article (10.1186/s12906-019-2590-9) contains supplementary material, which is available to authorized users.

## Background

Colon cancer is the second most common cause of cancer-related death following lung cancer [1, 2]. However, clinical therapeutic approaches for treating colon cancer are still limited to surgical resection, radiation and chemotherapy [3–5]. For that reason, scientists have continued to investigate additional targeted therapeutic strategies [6]. The present study was based on these principles and aimed to identify small molecules that specifically induce cell cycle arrest at G0/G1 phase and trigger the cellular apoptotic response in cancerous cells.

Apiole, 1-allyl-2,5-dimethoxy-3,4-methylenedioxybenzene, also referred to as parsley apiol or simply apiol, is a phenylpropene derivative purified from various natural sources, such as the fruits of *Petroselinum crisp* [7], the seeds of *Enterolobium contortisiliquum* (leguminosae) [8], wild-growing *Salvia aegyptiaca* [9], and the leaves of *Cinnamomum verum Presl* [10], caraway (*Carum carvi L.*) [11] and *Pituranthos chloranthus ssp.* [12]. Medicinally, it has been used to treat menstrual disorders and as an abortifacient in ancient times. We previously studied the molecular mechanisms of apiole-induced G0/G1 cell cycle arrest and subsequent induction of apoptosis specifically observed in human colon cancer cells (COLO 205 cells) [13]. G0/G1 cell cycle arrest-related proteins, such as p53, p21 and p27, were upregulated, and the cyclin D1 protein was downregulated. In addition, we demonstrated that apoptotic cell death was induced by apiole through activation of caspase (caspase 3,8,9)-mediated pathways. We found that bax/bcl-2 triggering signals were activated with significantly induced DNA laddering formation and induction of a subG1 peak observed by flow cytometry analysis. While the detailed mechanism of the apiole-induced anti-proliferative effects in COLO 205 cells requires further investigation, our findings clearly indicate that apiole is a candidate compound for the development of clinical anticancer drugs.

Considering the aforementioned findings, along with the crucial goal of identifying more specific anti-cancer drugs, three apiole derivatives (AP-02, AP-04, and AP-05) were either chemically synthesized or commercially procured and subsequently evaluated for their anti-proliferative activity.

## Methods

### Chemical synthesis of AP-02 and AP-04 (Additional file 1)

**Fig. 1:**
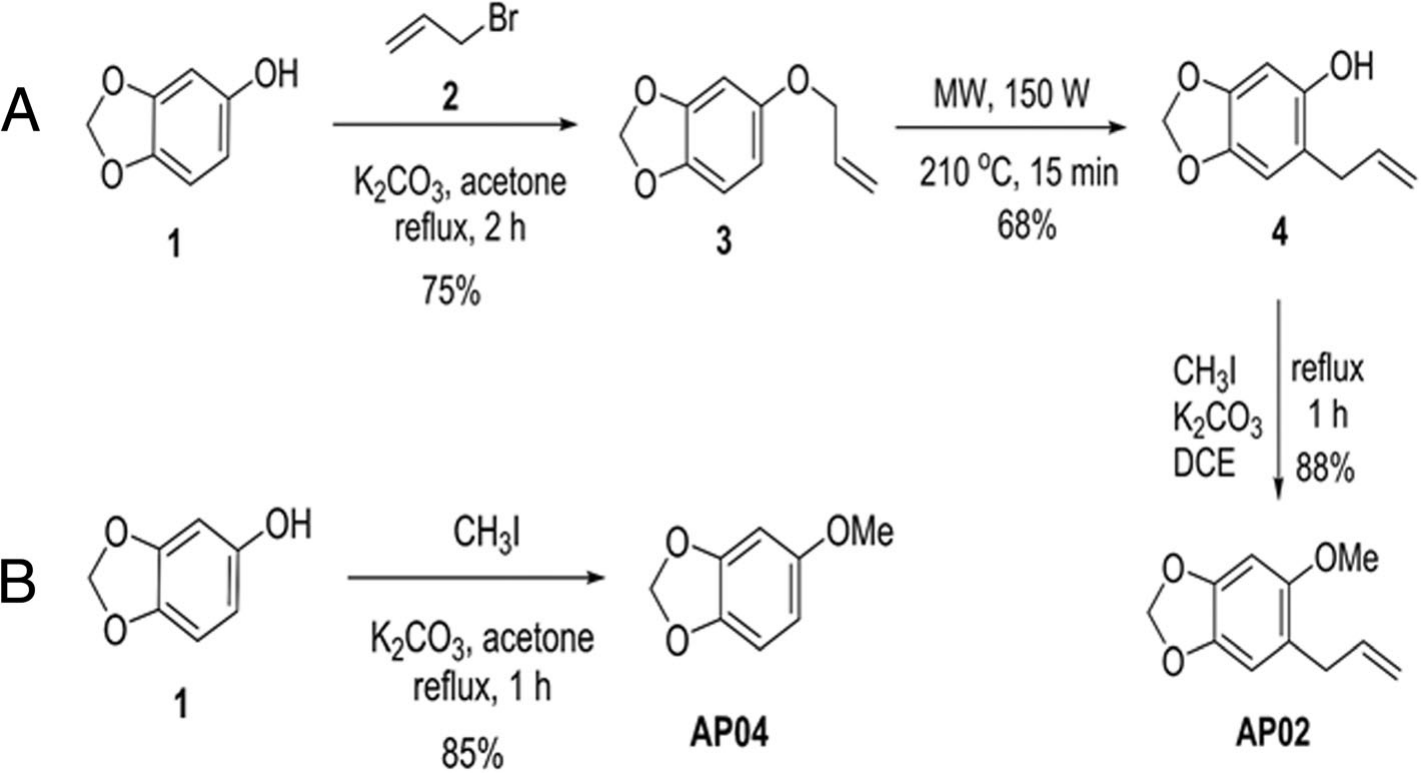
Chemical synthesis of apiole and its derivatives (**a** AP-02 and **b** AP-04)

Figure 1a depicts the preparation of AP-02. Preparation began with base-mediated O-allylation of commercially available benzo[d][1,3]dioxol-5-ol (1) with allyl bromide (2) in acetone under reflux conditions to give 5-(allyloxy)benzo[d][1,3]dioxole (3) at a 75% yield. The subsequent microwave-induced Claisen rearrangement of 3 yielded 6-allylbenzo[d][1,3]dioxol-5-ol (4) at 68% yield. The final O-methylation of 4 with methyl iodide in the presence of potassium carbonate in dichloroethane under reflux conditions afforded the target compound, AP-02, at 88% yield.

**Fig. 2:**
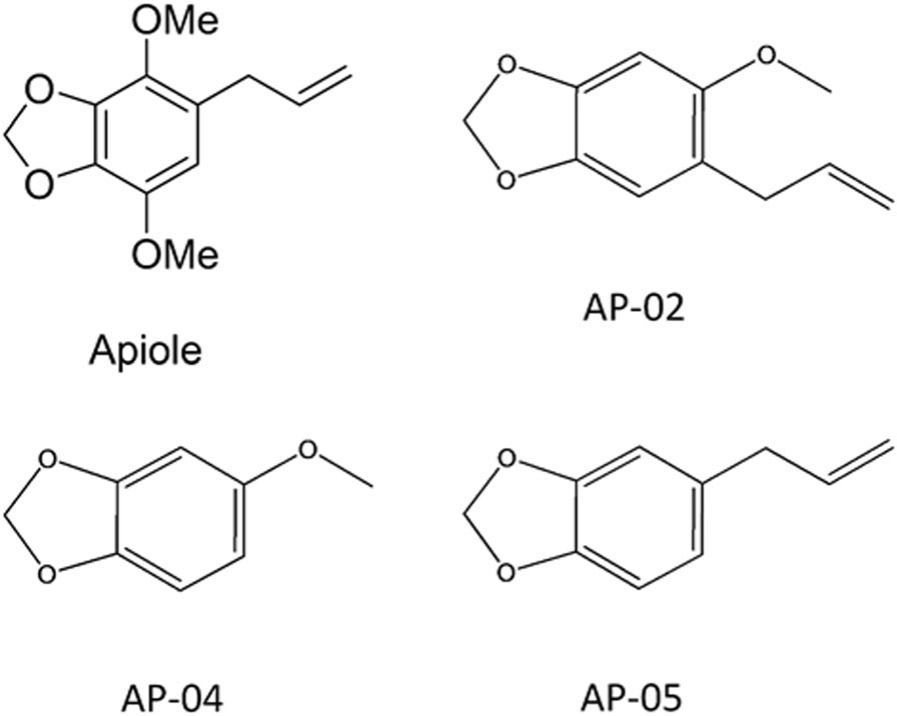
Chemical structures of apiole derivatives (AP-02, AP-04 and AP-05)

Figure 1b shows preparation of AP-04, which was readily available via base-mediated O-methylation of commercially available benzo[d][1,3]dioxol-5-ol (1) with methyl iodide in dichloroethane under reflux conditions for 1 h to give 5-methoxybenzo[d][1,3]dioxole (AP-04) at 85% yield. AP-05 was purchased from Acros Co. (Geel, Belgium, Cn). The chemical structures of apiole derivatives (AP-02, AP-04, and AP-05) is summarized in Fig. 2.

### Cell lines and cell culture

Human breast (ZR75, MDA-MB-231), lung (A549, PE089), colon (COLO 205, HT 29) and hepatocellular (Hep G2, Hep 3B) cancer cells were used in this study. Normal cells were used as controls (human breast (MCF 10A), lung (HEL 299), liver (BNL CL.2, Clone 9), and colon (FHC) cells) and were treated with the same regimens. MDA-MB-231 and ZR-75 cells were derived from human mammary gland and from a metastatic site of pleural effusion, respectively (American Type Culture Collection, ATCC® HTB-26™ and CRL-1500™). A549 cells were derived from human alveolar basal epithelial cell adenocarcinoma (ATCC® CCL-185™ and CRL-1500™). PE089 cells were isolated from a female patient with lung adenocarcinoma with an EGFR exon 19 deletion (courtesy of K. J. Liu from the National Health Research Institute). COLO 205 and HT 29 cell lines were isolated from human colon adenocarcinoma (ATCC® HMIC-38™ and CCL-222™). Hep 3B and Hep G2 cell lines were derived from human hepatocellular carcinoma (ATCC® HB-8064™ and HB-8065™) (Knowles et al., 1980). MCF-10A cells were isolated from normal human epithelial cells of the mammary gland (ATCC® CDR-10317™). HEL-299 cells are human embryonic lung cells derived from embryonic lung tissue (ATCC® CCL-137™). BNL CL.2 is a normal murine liver cell line (ATCC® TIB-73™). Clone 9 is a normal rat liver epithelial cell line (ATCC® CRL-1439™). FHC cells are normal human colon epithelial cells (ATCC® CRL-1439™). The p53 gene in both Hep G2 and COLO 205 cells is wild type [14–16], whereas p53 is partially deleted (7 kb) in the Hep 3B cells and mutated (codon 273) in the HT 29 cells [14, 17]. Cells were cultured in Eagle’s Minimal Essential Medium (for Hep 3BHep G2, and PE089 cells), Minimal Essential Medium (for HEL-299 and Clone 9 cells) or RPMI 1640 (for COLO 205, HT 29, FHC and A549 cells) supplemented with 50 μg/ml gentamycin, 0.3 mg/ml glutamine and 10% fetal calf serum (FCS). A 3:1 mixture of Ham’s F12 medium and DMEM (for MCF-10A cells) was supplemented with 10% FCS, 40 ng/ml hydrocortisone, 0.01 mg/ml cholera toxin, 0.005 mg/ml insulin, and 10 ng/ml epidermal growth factor. The BNL CL.2 cell line was selected to grow in medium containing ornithine and phenylalanine in place of arginine and tyrosine.

### Determination of cell viability

Cells were treated with compounds (Apiole, AP-02, AP-04, and AP-05) in a dose range from 4.5 to 600 μM for 24 h, and IC50 values were determined. Cell viability was determined at indicated apiole doses using 3-(4,5-dimethylthiazol-2-yl)-2,5-diphenyl-2H-tetrazolium bromide (MTT) assay. Briefly, cells were seeded in 96-well plates at a density of 1 × 10^4^ cells/well overnight. Next, medium in each well (200 μL) was replaced with 10 mmol/L HEPES (pH 7.4), and MTT dye (50 μL) was added to each well. Plates were incubated for 2~4 h at 37 °C in the dark. Media was removed and replaced with 200 μL phosphate-buffered saline (PBS) and 25 μL Sorensen’s glycine buffer. The absorbance ratio was measured at 570 nm using an ELISA plate reader.

### Cell synchronization, drug treatments, and flow cytometry analysis

Cells were washed with PBS three times 24 h after plating and incubated with media containing 0.04% fetal calf serum (FCS) for an additional 24 h. Under these conditions, cells are arrested in G0/G1 phase based on flow cytometry analysis as reported in our previous study [18]. Next, synchronized cells (cultured in 0.04% FCS) were challenged with the addition of media containing 10% FCS. Apiole- and PBS-treated groups were assessed via flow cytometry analysis to determine cell cycle distribution. Cells were stained with propidium iodide (50 μg/ml) (Sigma Chemical Co., St. Louis, MO, USA), and DNA content was assessed using a FACScan laser flow cytometry analysis system (Becton-Dickinson, San Jose, CA, USA), with 15,000 events being analyzed for each sample.

### Western blot analysis

Cell lysates were prepared, electrotransferred, immunoblotted with antibodies, and then visualized via incubation with colorigenic substrates (nitroblue tetrazolium, NBT, and 5-bromo-4-chloro-3-indolyl phosphate, BCIP) (Sigma Chemical Co., St. Louis, MO, USA). Expression of GAPDH was used as a control to ensure equal protein loading [19].

Immunodetection was performed by probing with appropriate dilutions of specific antibodies at room temperature for 2 h. Anti-p53, anti-p27/Kip1, anti-p21/Cip1, anti-GAPDH monoclonal antibodies (Santa Cruz, Inc. CA, USA), anti-E cadherin [SP64] (ab227639) (Abcam Inc. Shanghai, China), and anti-cyclin D1 monoclonal antibodies (Transduction Laboratories, Lexington, KY) were used. Membranes were incubated with secondary alkaline phosphatase-coupled anti-mouse and anti-rabbit antibodies (Jackson, Westgrove, PA, USA) at room temperature for 1 h at dilutions of 1:5,000 and 1:1,000, respectively. Immunoreactive proteins were visualized with a chemiluminescent detection system (PerkinElmer Life Science, Inc., Boston, MA, USA) and BioMax LightFilm (Eastman Kodak Co., New Haven, CT, USA) according to the manufacturer’s instructions. Results were analyzed by densitometry analysis.

### Animals

Four-week-old athymic nude mice were purchased from the National Science Council Animal Center, Taipei, Taiwan. Five animals in each cage were fed and acclimatized in our experimental animal center for 2 weeks in rectangular cages, and a small wood strip served as environmental enrichment. Animals were fed with the lab diet 5 k52 formulation (6% fat), and water was accessible at all times. Our animal facility was maintained under standard specific-pathogen-free conditions and a 12 h/12 h light/dark cycle. The total number of animals used in this experiment was 21, and the animals were equally divided among the 3 groups based on tumor volume. Sample size calculation was considered as follows: effect size = total number of animals − total number of groups, and the number of animals in each experimental group was *n* = 7. If the tumor size was greater than 4 cm^3^ or the mouse weight was 15% below the original weight, euthanasia was performed by placing the mice in a chamber and piping in carbon dioxide (CO_2_) at increasing concentrations until the animals became unconscious and died.

### Treatment of COLO 205-derived xenografts in vivo

COLO 205 cells were cultured in media as described above. Cells (5 × 10^6^) were suspended in 0.2 ml medium and injected subcutaneously between the scapulae of nude mice (purchased from National Science Council Animal Center, Taipei, Taiwan). For determination of tumor growth, the tumor volume was measured according to the following formula: tumor volume (mm^3^) = *L* x *W*^2^/2, where *L* is the length and *W* is the width [19]. Once tumors reached a mean size of 200 mm^3^, experimental animals were treated with either 25 μl PBS or 1 to 5 mg/kg AP-02 via intraperitoneal injection three times per week for 6 weeks. Control animals were treated with PBS at the same volume.

### Statistical analysis

For each analysis, data are represented as the mean ± SEM of at least three independent experiments. For comparison, statistical significance was tested using t-tests. All *p*-values were based on two-sided statistical analyses, and *p* < 0.05 was considered statistically significant.

## Results

### The Ap-02 apiole derivative preferentially induces cytotoxicity in human colon cancer cells

**Fig. 3:**
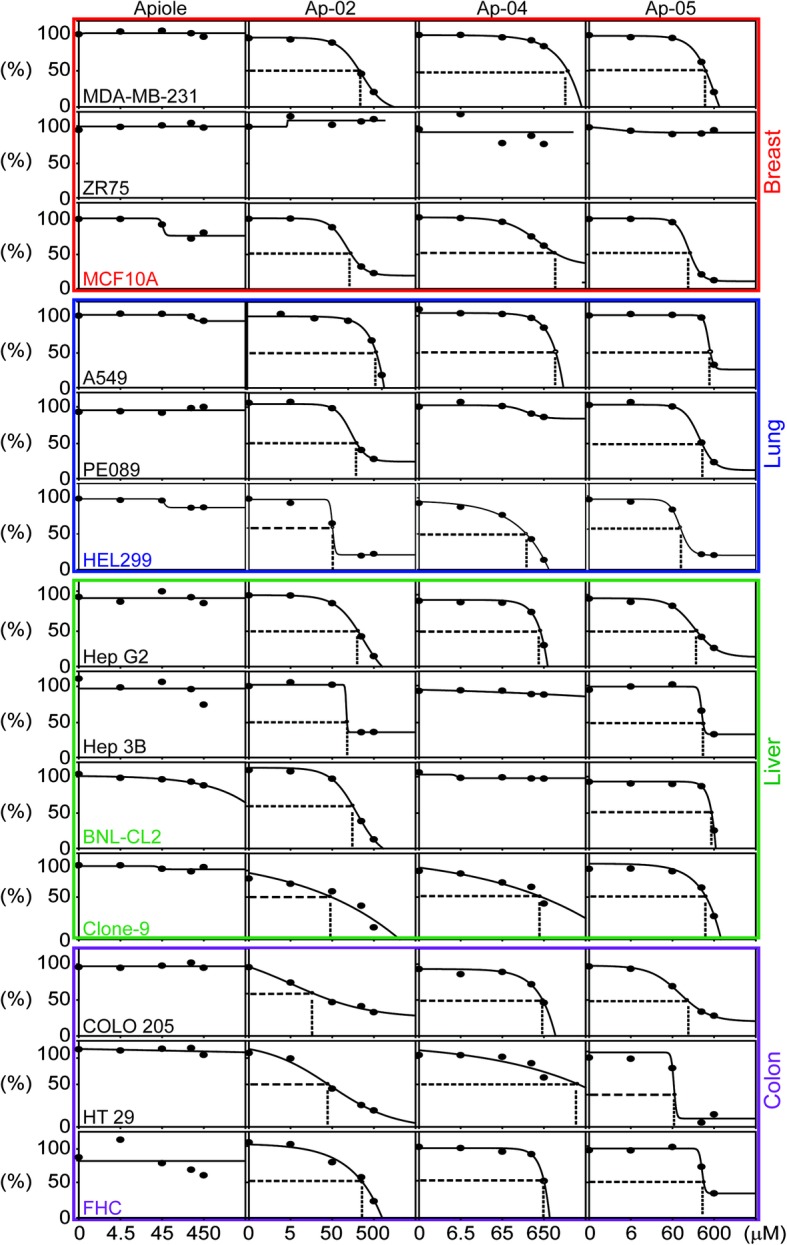
Cytotoxicity of apiole and its derivatives (AP-02, AP-04 and AP-05) in human cancer cells and normal cells. In this study, human breast (MDA-MB-231, ZR75), lung (A549, PE089), liver (Hep G2, Hep 3B), and colon (COLO 205, HT 29) cancer cells, as well as normal human breast (MCF 10A), lung (HEL 299), liver (BNL-CL2, Clone 9), and colon (FHC) cells were treated with compounds (apiole, Ap-02, Ap-04, and Ap-05; 4.5–600 μM) for 24 h, and IC50 values were determined

**Fig. 4:**
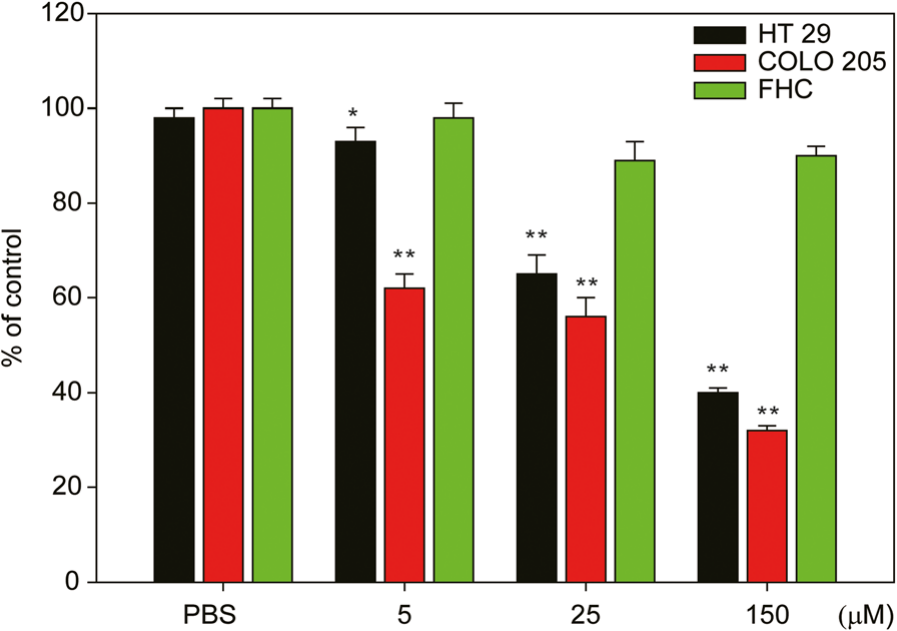
Dose-dependent response of the cytotoxic effects of human colon cancer and normal cells treated with AP-02. Viability of HT 29 and COLO 205 human colon cancer cells, as well as that of FHC (normal colon epithelial) cells, was evaluated after treatment with various concentrations of AP-02 (5–150 μM) for 12 h. Three samples were repeated in each group, and results are shown as the means ± SE

In this study, apiole and its derivatives (AP-02, 04, 05) were evaluated for their antitumor activities. To determine whether these compounds induce cell death in human cancer cells derived from different organs, we selected human breast (ZR75, MDA-MB-231), lung (A549, PE089), colon (COLO 205, HT 29) and liver (Hep G2, Hep 3B) cancer cells and treated cells with each compound (apiole, Ap-02, Ap-04, and Ap-05; 4.5–600 μM) for 48 h. IC50 values were then determined (Fig. 3 and Table 1). Normal human breast (MCF 10A), lung (HEL 299), liver (BNL CL.2, Clone 9), and colon (FHC) cells were treated in the same manner and served as controls. Among tested compounds, AP-02 was the most effective drug for inhibiting the proliferation of cancer cell lines compared to AP-04 and AP-05 (Table 1). We further observed that the cytotoxic effects of AP-02 were effective against human colon cancer cells (COLO 205 and HT 29, IC50 = 16.57 and 38.45 μΜ, respectively) compared to normal colon (FHC, IC50 = 263.76 μΜ) cells (Table 1, **p < 0.01). Interestingly, the cytotoxicity of AP-02 in normal colon (FHC) epithelial cells was reduced compared to other normal cells derived from the breast, lung and liver (MCF-10A, HEL299, BNL CL.2, and Clone 9, respectively). These results imply that compared to AP-04 and AP-05, AP-02 is valuable for targeting colon cancer cells (Table 1 and Fig. 3, **p < 0.01). Colon cancer cell lines (HT 29 and COLO 205) and normal (FHC) cell line were then treated with AP-02 across a range of doses (5–150 μM, for 24 h). Results indicated that AP-02 was the most effective compound with respect to induction of cytotoxicity, specifically in COLO 205 cells (Fig. 4, red bars, **p < 0.01).

**Table 1:** Cytotoxic effects of Apiole derivatives (AP-02, 04, 05) upon various human breast (MDA-MB-231, ZR75), lung (A549, PE089), liver (Hep G2, Hep 3B), and colon (COLO 205, HT 29) cancer cells

Organ	Tissue Sources	Cell lines	Time (h)	Mean IC_50_ ± SD (μM)			
				Apiole	AP-02	AP-04	AP-05
Breast	Human breast cancer	MDA-MB-231	48	NA	227.27 ± 2.90	NA	380.20 ± 3.28
	Human breast cancer	ZR-75	48	NA	NA	NA	NA
	Human breast epithelial	MCF-10A	48	NA	138.19 ± 2.31	NA	158.66 ± 1.92
Lung	Human lung cancer	A549	48	NA	348.97 ± 2.62	NA	486.03 ± 4.27
	Human lung cancer	PE089	48	NA	191.79 ± 2.30	NA	300.66 ± 2.48
	Human lung fibroblast	HEL299	48	NA	50.95 ± 0.46	241.44 ± 3.90	91.85 ± 1.25
Liver	Human liver cancer	HepG2	48	NA	206.03 ± 7.37	526.81 ± 0.81	225.33 ± 4.37
	Human liver cancer	Hep3B	48	NA	122.92 ± 2.59	NA	322.50 ± 1.47
	Rat liver epithelial	Clone 9	48	NA	43.67 ± 0.95	513.89 ± 16.47	374.41 ± 3.95
	Mouse liver epithelial	BNL cl.2	48	NA	153.58 ± 0.54	NA	522.62 ± 3.55
Colon	Human colon cancer	Colon 205	48	NA	16.57 ± 0.39	604.34 ± 19.80	133.77 ± 1.32
	Human colon cancer	HT-29	48	NA	38.45 ± 1.85	NA	67.65 ± 0.06
	Human colon epithelail	FHC	48	NA	263.76 ± 6.31	263.76 ± 4.63	336.47 ± 6.03

NA: IC _50_ non-available, ^*^ *p* < 0.05; ^**^ *p* < 0.01

### Low concentrations of Ap-02 inhibit human colon cancer cell proliferation through induction of G0/G1 cell cycle arrest

**Fig. 5:**
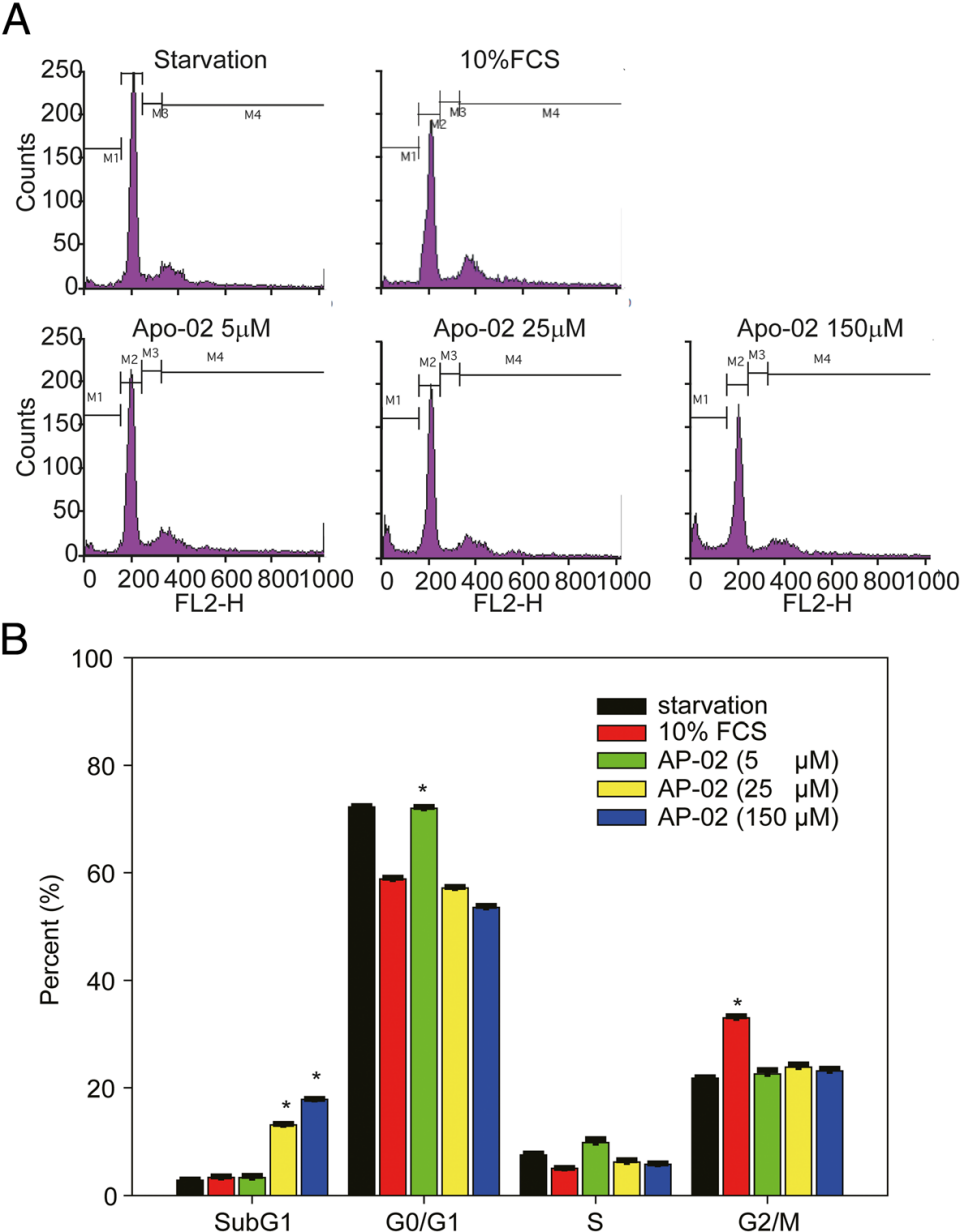
AP-02 induces G0/G1 phase cell cycle arrest and apoptosis in COLO 205 human cancer cells. **a** Before cell cycle experiments, COLO 205 cells were synchronized using 0.04% FCS for 24 h as described in Materials and Methods (upper left). After serum starvation, synchronized cells were replenished with complete medium (10% FCS) containing PBS (upper right) or 5, 25, or 150 μM AP-02 in PBS (lower panel). FACS analysis of DNA content 15 h after release from quiescence through incubation in culture medium supplemented with 10% FCS and various concentrations of AP-02 in PBS. **b** Percentages of cells in the sub-G1, G0/G1, S, and G2/M cell cycle phases were determined using CellFIT DNA analysis software. Three samples were analyzed in each group, and values represent the mean ± SE

Based on the results described above, we next investigated the specific mechanisms of AP-02 against cancer cells and selected COLO 205 cells as an in vitro cell model to test for cell cycle inhibition effects using flow cytometry (Fig. 5). We synchronized COLO 205 cells by culturing them in 0.04% FCS medium for 24 h according to our previous studies [18, 20]. Synchronized cells were than treated with 10% FCS to reactivate cell proliferation (Fig. 5, red bars). Our previous studies [18, 20] showed that the major differences in G0/G1 cell populations between serum starved and reactivated (10% FCS-treated) COLO 205 cells were observed 15 h after the media was replenished with complete media (Fig. 5a). COLO 205 cells in the PBS-treated group were in S phase at this time point (15 h) [20, 21]. According to these data, we selected this time point (15 h) to test AP02-induced G0/G1 arrest effects across a range of doses. The minimal dose of AP-02 that induced G0/G1 arrest was evaluated via flow cytometry analysis (Fig. 5b). Results indicated that the minimal dose of AP-02 needed to induce significant G0/G1 arrest was 5 μΜ (Fig. 5b, green vs. red bars, *p < 0.05). We further demonstrated that higher doses of AP-02 (> 5 μM for 24 h) induced appearance of a significant subG1 phase cell population (Fig. 5b, yellow and blue bars, *p < 0.05). These results indicate that AP-02-induced cancer cell death was occurring in high-dose groups.

### Ap-02 induces antitumor effects in vivo in COLO 205-xenograft tumors

**Fig. 6:**
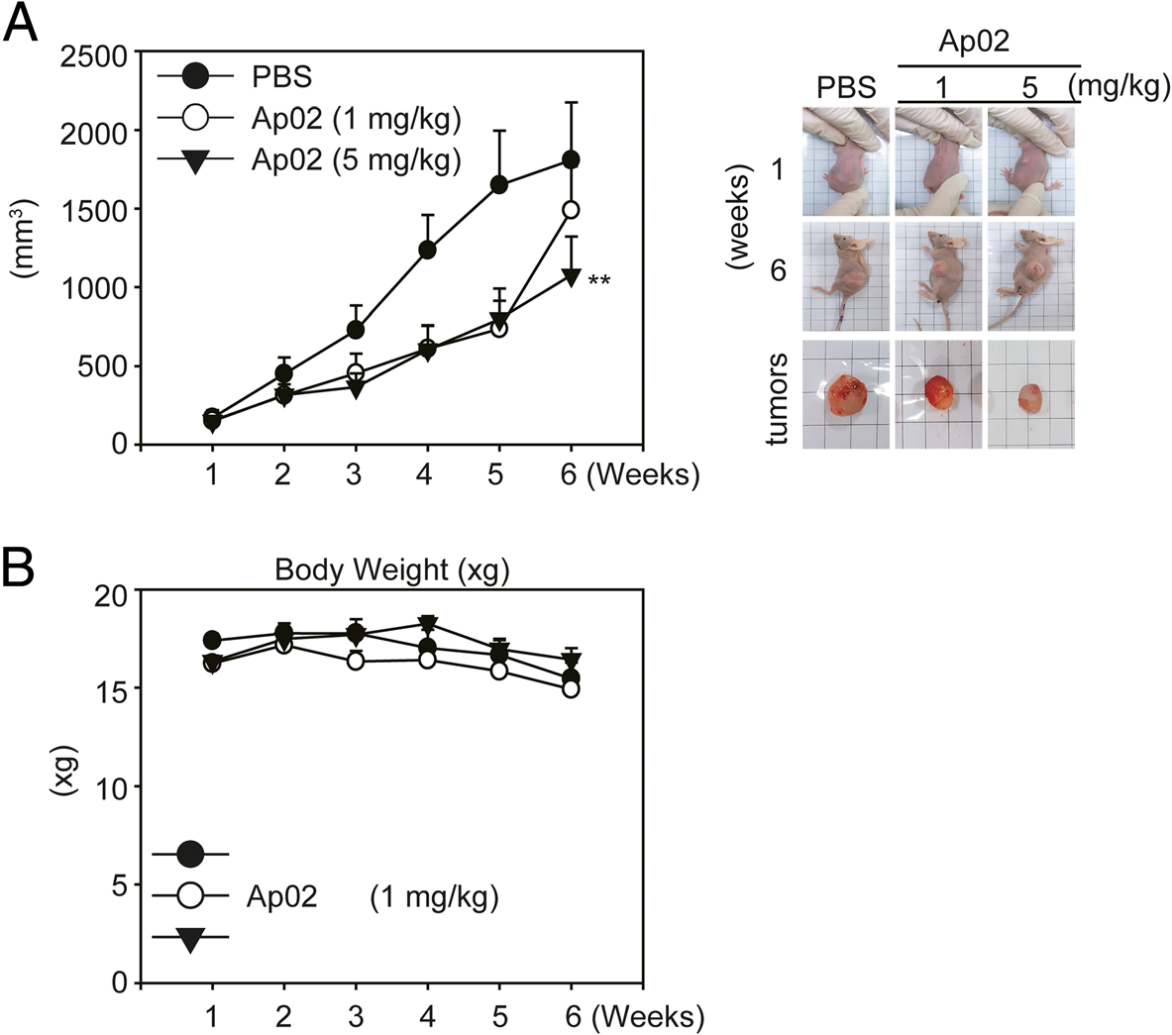
Growth of COLO 205 tumor xenografts in nude mice is suppressed in response to AP-02 treatment. Athymic nude mice were injected with COLO 205 cells into interscapular subcutaneous tissue. Once tumor volume reached approximately 200 mm^3^, animals were treated with 1 or 5 mg/kg AP-02 or PBS intraperitoneally three times per week for 6 weeks. **a** Average tumor volume of PBS-treated (circle, *n* = 5) versus AP-02-treated (*n* = 5) nude mice. **b** Body weight was measured each week during the experiment. Five samples were analyzed in each group, and values represent the means ± SE. Comparisons were subjected to Student’s *t*-test. ^*^ Significant difference at *p* < 0.05

To evaluate AP-02-induced antitumor effects, we used athymic nude mice bearing COLO 205 tumor xenografts as an in vivo animal model. Palpable tumors were established (mean tumor volume, 200 mm^3^). Next, COLO-205 tumor xenograft animals were treated with AP-02 (1 and 5 mg/kg, I.P. injection) or treated with the same volume of PBS as a control. Results indicated that AP-02 (> 1 mg/kg) treatment significantly reduced tumor volume compared to the control group (Fig. 6). No gross signs of AP-02-induced drug toxicity were detected in body weight changes (Fig. 6b). The general and microscopic appearance of individual organ tissues was also assessed (data not shown).

### G0/G1 cell cycle and metastasis regulatory proteins are involved in Ap-02-induced antitumor effects in COLO 205-xenograft tumors

**Fig. 7:**
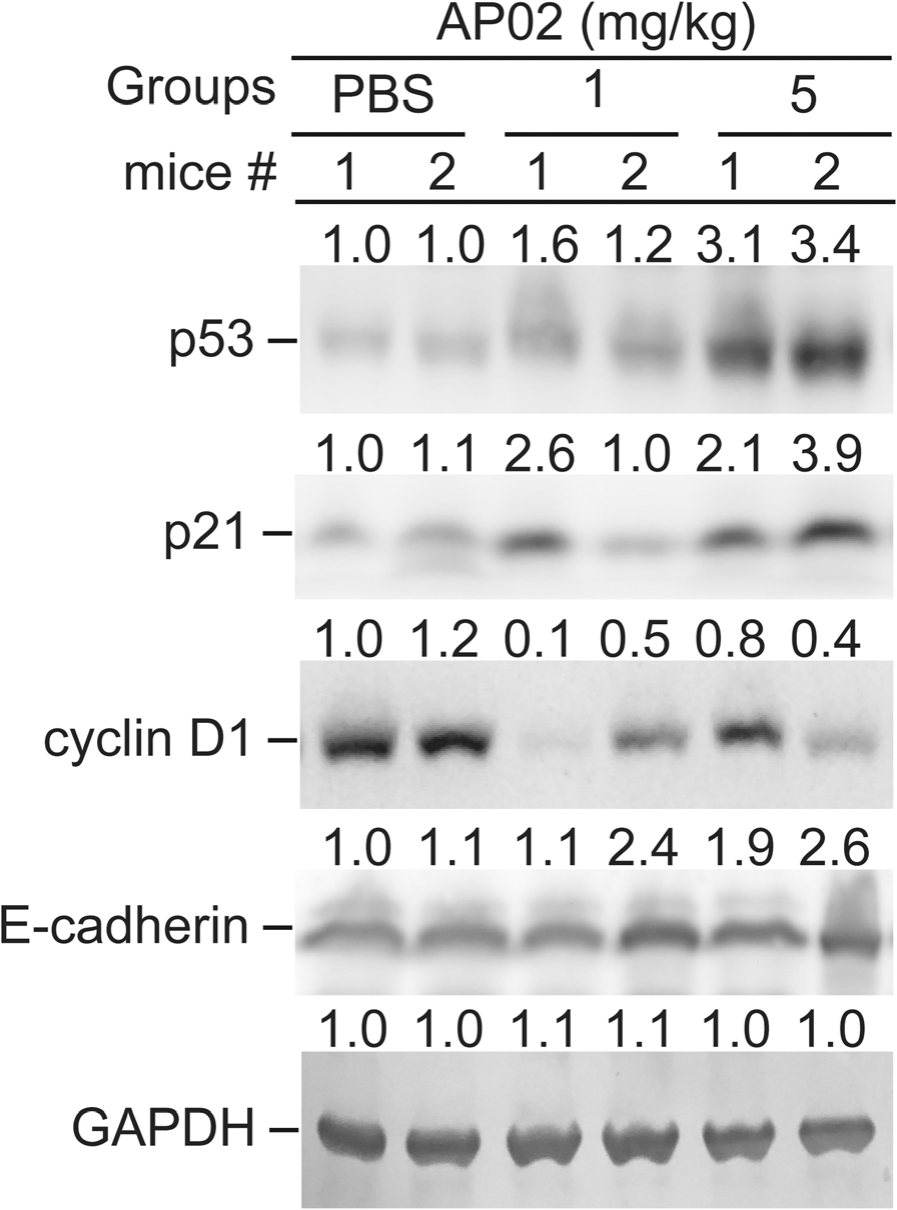
Effect of AP-02 on expression of G0/G1 phase regulatory and metastasis-related proteins in COLO 205 xenograft tumor tissues. Tumor tissues were dissected to isolate protein lysate extracts. Protein extracts (100 μg per lane) were separated via SDS-PAGE. After electrophoresis, proteins were transferred onto Immobilon-P membranes, which were probed with the proper dilution of specific antibodies. Proteins were then detected using the NBT/BCIP system. Membranes were also probed with anti-GAPDH antibody to correct for differences in protein loading. M, molecular weight marker. Representative data from two of seven tested animals are shown. The number below each line was detected by densitometry analysis and indicates the relative intensity of protein expression compared to the control-treated group (defined as 1 in mouse #1)

In this study, we found that high doses (> 25 μΜ) of AP-02 induced the appearance of a significant subG1 phase cell population in COLO 205 cells compared to the 10% FCS-treated group (Fig. 5b, *p < 0.05). However, in the lower dose Apo2-treated group (< 5 μΜ), only G0/G1 cell cycle arrest was detected (Fig. 5b, green bar, *p < 0.05). Based on these results, we suggest that G0/G1 cell cycle regulatory proteins are likely involved in AP-02-induced effects. We next confirmed these in vivo observations in AP-02-treated (1 and 5 mg/mL) COLO 205-xenograft tumor tissues. We found that expression of p53 and p21/Cip1 proteins was significantly induced, while cyclin D1 was downregulated in AP-02-treated tumor tissues (> 1 mg/kg) compared to controls (Fig. 7). To test whether AP-02 effectively inhibited metastasis-related signals in COLO 205-xenograft tumor cells, we selected E-cadherin, which functions as an invasion suppressor, as an indicator and determined whether it was upregulated in AP-02-treated tumors. We demonstrated that AP-02 treatment upregulates expression of E-cadherin protein (> 1 mg/kg) (Fig. 7). These results suggest that induction of E-cadherin by AP-02 inhibits COLO 205 cancer cell migration. The E-cadherin protein has also been used as a target for therapeutic purposes to prevent colorectal cancer cell metastasis [22]. Therefore, AP-02 may have clinical utility as a novel small molecule to prevent colorectal cancer cell metastasis.

## Discussion

*Petroselinum crispum* has been used as a medicinal herb. Plant extracts isolated from either the leaf or stem have been evaluated for antioxidant and DNA damage protection effects in normal (3 T3-L1) cells, in addition to their antitumor effects in breast cancer (MCF-7) cells [23]. Plant extracts from *Petroselinum crispum* have been investigated for different medicinal properties, including anti-mosquito properties [24] and anti-cadmium neurotoxicity in albino mice [25]. Toxicity and hematological studies in rats indicated that *Petroselinum crispum* was nontoxic at the applied dosage (< 1000 mg/kg) [26]. In this study, the antitumor effects of AP-02 derived from *Petroselinum crispum* were tested in human colon cancer cells. Interestingly, in vitro studies demonstrated that AP-02 administration induced significant G0/G1 cell cycle arrest in COLO 205 cells at concentrations less than 5 μΜ compared to the 10% FCS-treated group. However, this effect was not observed in normal human colon epithelial (FHC) cells. To examine the mechanism whereby these effects occur, an in vivo study was performed, which indicated that AP-02 affects G0/G1 phase regulatory proteins, significantly inducing p53 and p21/Cip1 protein expression and downregulating cyclin D1 expression compared to the control-treated group. Taken together, these results demonstrate that AP-02 may have potential for antitumor application through induction of cell cycle inhibition, specifically G0/G1 phase arrest, in colon cancer cells.

According to previous studies, apiole has been isolated from many plants [8, 27]. For example, *Petroselinum crispum* [27], which is used as a culinary herb and widely used as a seasoning condiment, is in the Apiaceae family. In this study, the antitumor effects of apiole derivatives (AP) were tested in different human cancer cell lines. Interestingly, human COLO 205 colon cancer cells were the most sensitive cells in responding to Ap-02 treatment. Another study further demonstrated that *Petroselinum crispum* extract induces cytotoxicity in hepatocellular carcinoma (Hep G2) cells [28]. Another study demonstrated that extract isolated from the seed of *Enterolobium contortisiliquum* exerts anti-migratory effects on gastric cancer cells [29, 30]. Extract from the leaves of *Cinnamomum verum Presl* [8] demonstrated antitumor effects in human colon cancer cell lines through targeting topoisomerase 1 and 2 [31, 32]. These studies all reveal the prominent antitumor activity of these plant extracts. However, the components mediating these effects were not identified. Our study provides evidence indicating that apiole derivatives, which were detectable in these plant extracts, may have antitumor properties.

Interestingly, we found that AP-02 was the most effective at inhibiting COLO 205 cell proliferation compared to another colon cancer cell line, HT 29. We suggest that p53-mediated p21 upregulation is likely the major mechanism involved in AP-02-induced G0/G1 phase cell cycle arrest. The p53 protein is well known for induction of cancer cell growth cycle arrest, as well as for inducing apoptotic cell death [33]. Wild type p53-mediated p21/CIP1-activated G0/G1 arrest has been implicated as a major anti-proliferation factor in cancer cell responses to antitumor drugs [33, 34]. Similar results were observed in a previous study, which indicated that rosiglitazone, a clinically used anti-type-2 diabetes drug, reduced tumor metastasis in human cancer cells. The study demonstrated that rosiglitazone had a significantly increased cytotoxic effect in p53-wild-type HCT116 cells but not in mutant p53 HT-29 cells [35]. Inhibition of MDM2, a negative regulator of p53, in addition to MDMX, which stabilizes p53, has been revealed as an effective strategy for improving radiotherapy outcomes [36]. For clinical radiotherapy, validation of wild type p53 protein expression in cancer cells is suggested. A similar study demonstrated that upregulation of wild type p53 effectively activates an early-onset breast cancer-related gene (GAS7) and, through the GAS7-CYFIP1-mediated signaling pathway, effectively suppresses breast cancer metastasis [37]. These results suggest that natural compounds, such as AP-02, that inhibit early-stage tumor cell formation through the activation of wild type p53-mediated signals might be valuable for chemoprevention.

According to our previous study, we demonstrated that 4,7-Dimethoxy-5-methyl-l,3-benzodioxole (SY-1) which was isolated from fruiting body of Antrodia camphorate induced significant cell cycle arrest (50–150 μM) and apoptosis (> 150 μM) in colon cancer cells [38, 39]. The SY-1 derivative Apiole is also present in different types of natural plant products [40]. We further demonstrated that Apiole is a potential antitumor agent tested by in vivo animal study [13]. To test the dosage range of Apiole, we examined the therapeutic efficacy of apiole in vivo (1, 5, or 30 mg/Kg body weight respectively) 3 times per week for 30 days by treating athymic mice bearing COLO 205 tumor xenografts. No gross signs of toxicity were observed in the mice receiving the treatment regimens. After 30 days, the tumor growth in the groups treated with doses higher than the 1 mg/Kg body weight apiole was significantly inhibited relative to the growth observed in the control-treated mice. In this study, the Apiole derivatives (AP-02) were tested for effective in vitro cytotoxicity (5 μM) and in vivo antitumor activity compared to the control group (> 1 mg/kg, *p < 0.05). The dosage is lower than the Apiole as described. These results may have relevance for colon cancer chemotherapy.

Our finding suggest that AP-02 inhibits cell cycle progression, which is regulated by successive, coordinated activation of CDKs [41]. We also found that AP-02 affects dysregulation of a series of regulatory subunits called cyclins and a group of CDK-inhibitory proteins (CKIs) [42, 43]. Among these CKIs, the two most well known are p21/Cip1 and p27/Kip1. This study demonstrated that AP-02 effectively induces p21/Cip1and p53 protein upregulation in COLO 205 xenograft tumors in vivo. p21/Cip1 expression may be induced by the p53 tumor suppressor gene in human cancer cells [44, 45]. In addition, an in vivo study further demonstrated that cyclin D1 protein levels were downregulated. These results suggest that activation of p21/Cip1 by AP-02 is associated with and inactivates CDKs, resulting in cell cycle arrest [43, 46]. In addition, E-cadherin has been reported to function as an invasion suppressor and is downregulated in most carcinomas. In contrast, N-cadherin is a reported invasion promoter that is upregulated in proliferative cancer cells. Upregulation of N-cadherin in epithelial cells induces morphological changes, including conversion to a fibroblastic phenotype, leading to malignant cancer cell phenotypes with motility and invasive characteristics. In our study, we found that after AP-02 treatment, E-cadherin protein was significantly upregulated (Fig. 7) compared to the control-treated group. These results suggest that AP-02 is a potential therapeutic agent for the prevention of tumor cell metastasis.

## Conclusion

Our study reveals the detailed mechanisms underlying AP-02-induced antitumor effect on COLO 205 xenograft tumor growth formation. Our experimental findings demonstrate that AP-02 inhibits cancer cell proliferation through G0/G1 cell cycle arrest (< 5 μM, low dose) in addition to apoptosis induction (high dose) in wild-type p53 colon cancer (COLO 205) cells, both in cultured cells and in xenograft animals. These results highlight the molecular mechanism by which AP-02 induces antitumor effects in colon cancer, which might have clinical relevance for future applications.

## Additional file

Additional file 1: Chemical synthesis of AP-02 and AP-04. (ZIP 6 kb)

## Data Availability

Readers can access original data and materials by email. Please contact the corresponding author Dr. Yuan-Soon Ho (email: hoyuansn@tmu.edu.tw).

## Abbreviations

AP: Apiole
BCIP: 5-bromo-4-chloro-3-indolyl phosphate
FCS: Fetal calf serum
MTT: 3-(4,5-dimethylthiazol-2-yl)-2,5-diphenyl-2H-tetrazolium bromide
NBT: Nitroblue tetrazolium
PBS: Phosphate buffered saline
